# A quick, easy and efficient protocol for extracting high-quality RNA from *Mycobacterium tuberculosis* using a spin column commercial kit

**DOI:** 10.1186/s13104-023-06424-w

**Published:** 2023-07-13

**Authors:** NE Mvubu, A. Salig, K. Moopanar, ASG Nyide, D. Govender, E. Mankayi

**Affiliations:** 1 Medical Microbiology, School of Laboratory Medicine and Medical Sciences, College of Health Science, Medical School, University of KwaZulu Natal, Private Bag X54001, Durban, 4000 South Africa; 2 Microbiology, School of Life Sciences, College of Agriculture, Engineering and Science, University of KwaZulu Natal, Durban, South Africa

**Keywords:** *M. tuberculosis*, RNA extraction, Commercial kit, Protocol

## Abstract

RNA extraction from *Mycobacterium tuberculosis* has been a historically challenging task for researchers due to the thick lipids associated with the cell wall of this “notorious” pathogen that is responsible for Tuberculosis (TB) outbreaks. Several studies have successfully extracted RNA from *M. tuberculosis* using a Trizol reagent combined with organic solvents. Recently, our laboratory has successfully extracted high quality total RNA using a commercial kit from clinical strains belonging to F15/LAM4/KZN, Beijing and F11 strain families and H37Rv laboratory strain by exploiting high speed homogenizer for cell lysis and spin columns for RNA purification. The quality and integrity of the extracted RNA was analyzed and confirmed through the Nanodrop, Bioanalyzer and RNA 3-(N-morpholino) propanesulfonic acid (MOPS) gel electrophoresis. Furthermore, to confirm the integrity of small RNA (sRNA) molecules due to their vulnerability to degradation, the RNA samples were converted to cDNA and sRNAs were amplified and confirmed through PCR. This detailed RNA extraction protocol proposes to carve a new path into TB transcriptome research without the use of organic solvent for downstream purification steps while yielding high quality RNA that can be used to understand *M. tuberculosis* transcriptome regulation.

## Introduction

Nucleic acid extraction from *Mycobacterium tuberculosis* has been history challenging due to the thick lipids associated with the cell wall of this etiological agent of TB disease. Due to high demand of DNA in TB molecular diagnosis assays, its extraction has been optimized since the early 1900s [1–5]. However, DNA extraction from *M. tuberculosis* has been evolving with new optimized protocols for high quality DNA that can be used in sequencing studies [6–8]. Recently, a spin column-based extraction kit has been used for extracting high quality DNA from variety of *M. tuberculosis* samples without the use of organic solvents [9].

RNA extraction using a Trizol method has been optimized for variety of samples [10, 11] including *M. tuberculosis* [12, 13]. Lysing the cells in Trizol stabilize the RNA, which is usually followed by solvent extraction and precipitation using cold alcohols. Thus, *M. tuberculosis* transcriptome studies [14–16] have exploited this extraction protocol for the past few decades with overwhelming success. However, a better extraction protocol is needed that bypass the use of carcinogenic organic solvents without compromising the stability and quality of the extracted RNA. A few studies [17, 18] have explored commercial kits for total RNA extraction from *M. tuberculosis*, however, the stability of non-coding RNAs were not evaluated in these methods. The current protocol provides a detailed extraction method of high-quality total RNA from laboratory and clinical strains *M. tuberculosis* using a commercial spin-column kit. Furthermore, the extracted total RNA using this protocol can be used to study regulatory non-coding RNAs such as small RNAs (sRNAs) that can provide new insight into regulatory mechanisms of *M. tuberculosis* transcriptome.

## Materials and methods

The current study was approved by the Biomedical Research Ethics Committee (BREC) at the University of KwaZulu-Natal (reference no. BREC/00002909/2021). All methods were performed in accordance with the relevant guidelines and regulations from the University of KwaZulu-Natal BREC. *M. tuberculosis* Beijing, F11 and F15/LAM4/KZN (KZN) strains were isolated from sputum specimens of patients in the province of KwaZulu-Natal, South Africa [19] and the informed consent was obtained from all subjects and/or their legal guardian(s) during sample collection. The laboratory strain, *M. tuberculosis* H37Rv (ATCC 25,618) used as a virulent control was obtained from the culture collection in Department of Medical Microbiology and Infection control, University of KwaZulu-Natal (UKZN) South Africa. Strain identity was confirmed by means of IS6110-restriction fragment length polymorphism analysis (RFLP). Drug-resistance profiling confirmed that F15/LAM4/KZN and F11 were a representative of XDR-TB, while the Beijing and H37Rv represented MDR-TB and drug-sensitive TB, respectively. All *M. tuberculosis* strains were resuscitated from storage at -80 °C, thawed at room temperature, and cultured in Middlebrook 7H9 broth supplemented with 0.5% glycerol and 0.05% Tween-80 and 10% Oleic acid Albumin Dextrose Catalase (OADC) at 37 °C to an OD_600nm_ of 0.8-1 (1 × 10^8^) colony forming units (CFU/mL) [20].

To encourage the expression of sRNAs implicated to growth and cholesterol metabolism, the laboratory H37Rv strain was cultured in an in vitro cholesterol model using the protocol described by Pandey and Sassetti [21] and Chang, Miner [22] with the following composition per litre; 0.5 g asparagine, 1 g KH2PO4, 1.5 g Na2HPO4, 10 mg MgSO4.7H2O, 0.5 mg CaCl2, 0.1 mg ZnSO4, 50 mg ferric ammonium citrate, and 1 mL 1:1 (vol/vol) tyloxapol-ethanol containing 0.01% cholesterol. *M. tuberculosis* cells were initially grown in standard Middlebrook 7H9 media and pelleted when the log phase OD (0.8-1 _600 nm_) was achieved. The cells were thereafter washed twice and resuspended with minimal media that was deficient in supplemented cholesterol. This resuspension was used as the inoculum for the cholesterol-rich media, to a growing final volume of 100 mL. This was followed by total RNA extraction as described in detail:

### RNA extraction

#### ***M. tuberculosis*** lysis

1. Spin 100 mL of culture (OD ~ 0.8-1) into two 50 mL tubes for 15 min at 1481 Relative Centrifugal Force (RCF).
2. Discard supernatant.
3. Resuspend pellet in 1 mL TriZol reagent.
4. Transfer 1 mL of sample into an O-ring tube.
5. Add ~ 300 µL of zirconia beads to the O-ring tube.
6. Balance bead beater, press protocol 1 (3366 RCF for 30 s X3) and accept (Precellys 24 Dual Tissue Homogenizer was used. Low speed Disruptor Genie was also used at maximum speed for 30 min).
7. Keep on ice for 2 min.
8. Repeat step 6 and 7 two times (only for Precellys 24).
9. Centrifuge samples for 1 min at 17,640 RCF.
10. Transfer ~ 800 uL of supernatant to a 2 mL Eppendorf.

### RNA purification

#### Protocol (Zymogen Direct-Zol kit, cat # R2052)

11. Add 1 mL of molecular grade ethanol (95–100%) and mix thoroughly.
12. Transfer 1 mL of mixture to a **Zymo-spin IIICG Column** in a **Collection Tube** and centrifuge at 17,640 RCF for 30 Sec. 
13. Discard flow-through, add the rest of the mixture (~ 800 ul) and centrifuge at 17,640 RCF for 30 Sec. 
14. Transfer column into a **new** collection tube and discard flow-through.

NB: DNase I Treatment (Keep DNase on ice and store at -20°C)

15. Add 400µL **RNA Wash Buffer** to the column and centrifuge at 17,640 RCF for 30 Sec. 
16. In an RNase-free tube: add 35 µl **DNase I** (5 U/µl) and 75 µl **DNA Digestion buffer** per reaction and mix.
17. Add 80 µl of DNase I - DNA Digestion buffer mixture directly to the column.
18. Incubate at room temperature for 15 min.
19. Add 400 µL **Direct-Zol RNA PreWash** to the column and centrifuge at 17,640 RCF for 30 Sec. 
20. Discard flow-through and repeat the wash step (400 μL Direct-Zol RNA PreWash and centrifuge).
21. Add 700 µL **RNA wash buffer** to the column and centrifuge at 17,640 RCF for 2 min.
22. Transfer column to a 1.5 mL eppendorf.
23. Add 35 µl of **DNase/ RNase-free water** to column matrix and centrifuge at 17,640 RCF for 30 Sec to elute.
24. Optional: Repeat step 23 with the eluted 35 µL (do not add more DNase/RNase water because it will dilute the RNA concentration).
25. Make 10 μL aliquots into RNase free Eppendorfs to be used for MOPS gel and nanodrop analysis and keep the rest in -80°C to avoid freezing and thawing the RNA.

RNA quality was evaluated through 3-(N-morpholino) propanesulfonic acid (MOPS) gel electrophoresis following Good, Winget [23] recommendations. Briefly, 1% agarose gel was prepared by dissolving 1 g agarose in 72 mL distil water followed by addition of 10 mL of 10X MOPS (0.4 M MOPS powder (pH 7.0), 0.1 M sodium acetate and 0.01 M EDTA) buffer and 18 ml 37% formaldehyde (12.3 M). To completely denature RNA for gel electrophoresis 1–3 µg RNA was mixed with RNA loading dye and heated at 65 °C for 15 min prior to loading the gel followed by electrophoresis at 70 V for 90 min.

### cDNA synthesis and polymerase chain reaction

#### cDNA synthesis (Biorad iScript cDNA synthesis kit, cat #1,708,891)

Total RNA extracted from 7H9 broth and cholesterol rich minimal media were used for cDNA synthesis following iScript cDNA synthesis protocol. Briefly, RNA was initially heated at 70 °C for 10 min followed by immediate cooling on ice. Thereafter, RNA was reverse transcribed using the iScript cDNA Synthesis Kit containing 5x iScript Reaction Mix, iScript Reverse Transcriptase and nuclease-free water. The reaction was carried out in a thermal cycler (Bio-Rad) as follows: priming for 5 min at 25 °C, reverse transcription for 20 min at 46 °C and reverse transcriptase inactivation for 1 min at 95 °C. The cDNA samples were then stored at -20 °C and used for polymerase chain reaction (PCR).

### PCR

Eight primers, 4 sets (forward and reverse) (Table 1), were designed using SnapGene software (www.snapgene.com) (v6.1.1) for sRNAs linked to cholesterol metabolism in *M. tuberculosis* to evaluate their integrity using the modified Zymogen spin column extraction method. The Thermo Scientific DreamTaq DNA Polymerase kit (#EP0703) was used for amplification of sRNAs in the C1000 Touch™ Thermal Cycler (#) following initial denaturation of 95 °C for 3 min, denaturation at 95 °C for 30 s, primer specific annealing temperature (Table 1) for 30 s, extension at 72 °C 1 min for 35 cycles and final extension at 72 °C 10 min.

**Table 1:** sRNA primers that were used for PCR for H37Rv strain grown in cholesterol-rich minimal media

sRNA	Forward Primer (5’-3’)	Reverse Primer (5’-3’)	Expected product size (bp)	Annealing temperatures (°C)
*ASdes*	CATAGAGGACGGAGTCGG	AGTGGGTCAACCGTT	195	51
*ASpks*	GGCCTGCGTATGACCCATATT	CTTATGGGCAAGATCGGGGG	80	57
*MTS2823*	TAGTACAAAGGAACCAC	GACCCGAAGAACTCGA	300	55
*Mcr11*	AAAGTGCCGGAAGACGGG	CGGTACACATGGGCAGACCC	100	52

## Results and discussion

**Fig. 1:**
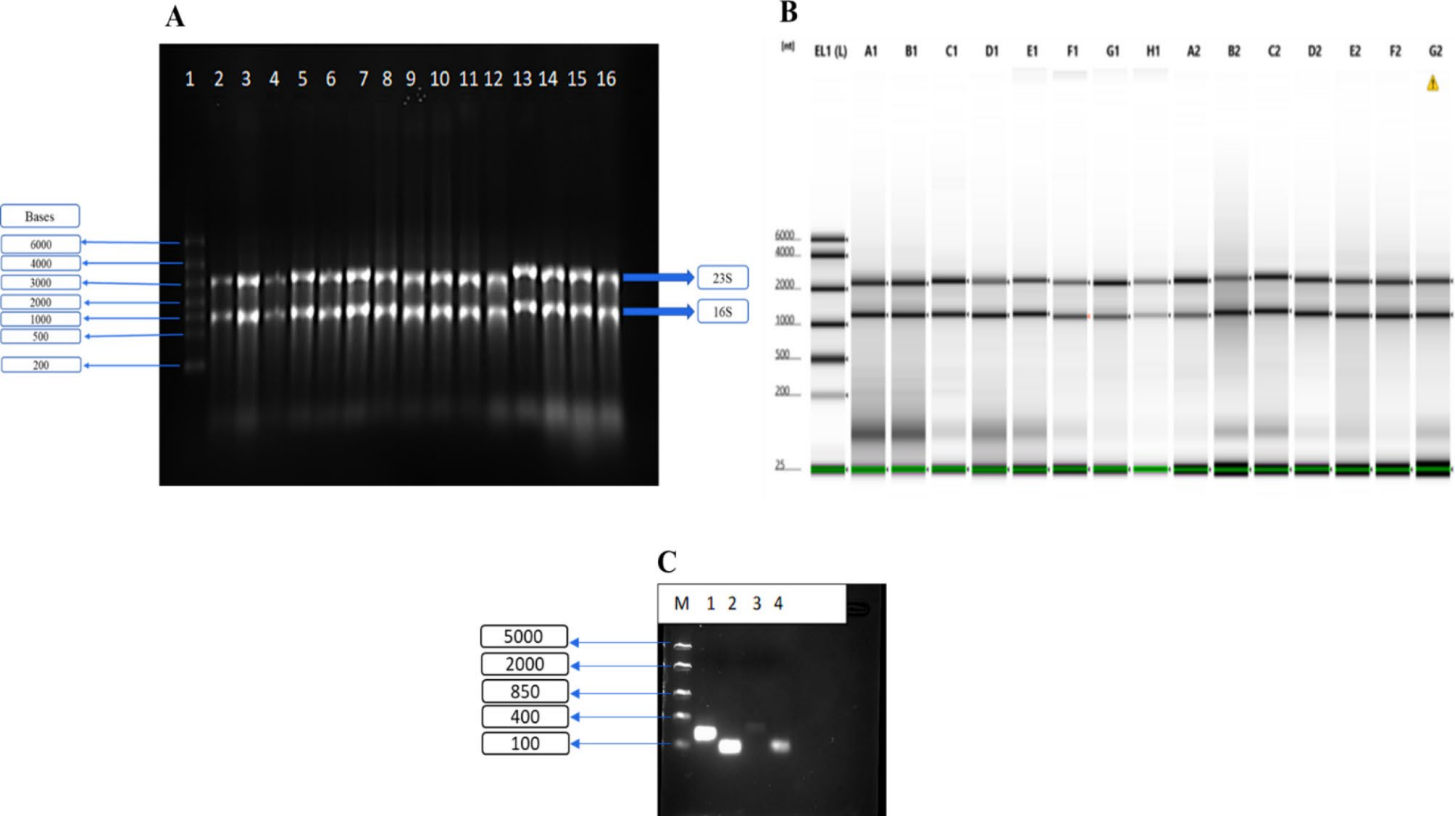
MOPS gel electrophoresis **(A)** and Bioanalyzer **(B)** showing the integrity of extracted RNA using a modified Spin column kit. The estimated sizes of 23 S and 16 S ribosomal subunits is 3000 bp and 1500 bp, respectively. Lanes in Fig. A: Lane 2–4 (KZN B1, B2 and B3), 5–7 (Beijing B1, B2 and B3), 8–10 (F11 B1, B2 and B3), 11–13 (H37Rv B1, B2 and B3), 14–16 (H37Rv Chol B1, Chol B2 and Chol B3); which correspond to Lane A1 to Lane G1, respectively in the Bioanalyzer image (shown in details on Table 2). **(C)** A 1.2% agarose gel showing PCR product for *ASdes* (Lane 1), *ASpks* (Lane 2), *MTS2823* (Lane 3) and *Mcr11* (Lane 4) for the expected product sizes of 195, 80, 300 and 100 bp, respectively, using the cDNA synthesized from the H37Rv laboratory strain cultured in minimal media supplemented with cholesterol. The agarose gel electrophoresis was running at 80 V for 50 min

The current protocol exploits the use of high-speed homogenizers and spin-column kit to extract good quality RNA from *M. tuberculosis*. RNA was successfully extracted from clinical strains of *M. tuberculosis* and a laboratory strain using a modified Zymogen spin-column kit. Shown in Fig. 1A is a MOP gel with intact large (23 S) and small (16 S) subunits around the expected sizes (3000 and 1500 bp), respectively. Furthermore, the integrity of the extracted RNA was confirmed through the Bioanalyzer at the correct size estimation (Fig. 1B) with their respective concentrations in Table 2. The extracted RNA was converted to cDNA followed by successful amplification of sRNAs as shown in Fig. 1C for *ASdes* (Lane 1), *ASpks* (Lane 2), *MTS2823* (Lane 3) and *Mcr11* (Lane 4) at 195, 80, 300 and 100 bp, respectively. The Nanodrop 260/280 and 260/230 ratios revealed no contamination of the extracted RNA with ratios between 1,88 − 2,17; and the Bioanalyzer RNA integrity number (RIN) was between 7,1 and 8.6, which can be used for further downstream application, including next generation sequencing (Table 2).

**Table 2:** Concentrations and purity of the extracted RNA samples from clinical and laboratory strains of *M. tuberculosis*

Sample ID	Nucleic Acid Conc. (ng/µl): Nanodrop	260/280	260/230	RIN: Bioanalyzer	MOPS Gel lane	Bioanalyzer Lane
F15/LAM4/KZN 7H9 B1	990.8	1.95	2	7.6	2	A1
F15/LAM4/KZN 7H9 B2	638.5	1.92	2.01	7.7	3	B1
F15/LAM4/KZN 7H9 B3	901.6	1.92	1.99	8.0	4	C1
BEIJING 7H9 B1	1296.1	1.93	2	8.2	5	D1
BEIJING 7H9 B2	1311.6	1.89	1.88	8.3	6	E1
BEIJING 7H9 B3	1213	1.95	2.08	8.1	7	F1
F11 7H9 B1	948.8	1.87	1.97	8.0	8	G1
F11 7H9 B2	614.1	1.96	2.07	7.2	9	H1
F11 7H9 B3	606.8	1.95	2.04	7.4	10	A2
H37RV 7H9 B1	561.2	1.88	1.87	7.2	11	B2
H37RV 7H9 B2	484.2	1.94	2.05	7.2	12	C2
H37RV 7H9 B3	1650.2	1.92	2.04	8.6	13	D2
H37RV Chol B1	1491.3	2.03	2.13	8.5	15	E2
H37RV Chol B2	1308.3	2.03	2.17	8.3	15	F2
H37RV Chol B3	870	2.04	2.15	7.1	16	G2

RIN: RNA integrity number

B: Biological replicate

7H9: Normal culture media used for *M. tuberculosis*

Chol: Minimal media supplemented with cholesterol

RNA isolation in *M. tuberculosis* has been successfully performed using a traditional Trizol-organic solvent extraction protocol over the years [14–16, 24]. Other studies [17, 25–27] have also exploited column-based extraction protocol from this notorious pathogen, with successful noncoding amplification [28]. The current study thus provides a detailed extraction protocol and tips for future researchers that are working on the *M. tuberculosis* transcriptome research.

This protocol exploit high speed homogenizer with microbeads and the commercially available spin-column kit to extract quality RNA samples that can be used in *M. tuberculosis* transcriptome studies. This protocol reduced the use of toxic organic solvents since it only requires Trizol and provides good yields and pure RNA samples. Furthermore, non-coding small RNAs extracted using this protocol can be used in sequencing studies to unravel complex mechanisms in gene regulation of this infectious pathogen; as well as transcriptome research as recently published by our team [29]. It should be noted that low speed homogenizers can be used for longer (~ 30 min) to successfully lyse *M. tuberculosis* followed by RNA purification and other commercial kits can be used, provided they are able to stabilize non-coding RNA.

### Limitations

The limitation in the current study is the need for the homogenizer, which can be a high-speed disruptor such as Precellys or low speed one such as the Disruptor Genie for the lysis of a thick cell wall of *Mycobacterium tuberculosis*.

## Data Availability

Not applicable.
